# Characterization of Primary Action Mode of Eight Essential Oils and Evaluation of Their Antibacterial Effect against Extended-Spectrum β-Lactamase (ESBL)-Producing *Escherichia coli* Inoculated in Turkey Meat

**DOI:** 10.3390/molecules27082588

**Published:** 2022-04-18

**Authors:** Chedia Aouadhi, Ahlem Jouini, Dhekra Mechichi, Mouna Boulares, Safa Hamrouni, Abderrazak Maaroufi

**Affiliations:** 1 Laboratory of Epidemiology and Veterinary Microbiology, Group of Bacteriology and Biotechnology, Pasteur Institute of Tunisia (IPT), Université de Tunis El Manar, 13 Place Pasteur, Belvédère, B.P. 74, Tunis 1002, Tunisia; ahlem.jouini@pasteur.tn (A.J.); mechichidhekra@yahoo.com (D.M.); safa.hamrouni@pasteur.tn (S.H.); abderrazak.maaroufi@pasteur.tn (A.M.); 2 Research Laboratory: Technological Innovation and Food Safety LR22-AGR01 École Supérieure des Industries Alimentaires de Tunis (ESIAT), Université de Carthage, 58 Avenue Alain Savary, Cité El Khadhra, Tunis 1003, Tunisia; boulares_mouna2006@yahoo.fr

**Keywords:** *E. coli*, essential oil, antimicrobial activity, action mode, turkey meat

## Abstract

The current study aims to evaluate the antimicrobial activity of eight essential oils (EOs) against multidrug-resistant *Escherichia coli* strains, producing extended-spectrum β-lactamase (ESBL) enzymes and isolated from foods. Disc-diffusion assay showed that the inhibition diameters generated by EOs varied significantly among the tested EOs and strains. In fact, EOs extracted from *Thymus capitaus*, *Eucalyptus camaldulensis*, *Trachyspermum ammi* and *Mentha pulegium* exerted an important antimicrobial effect against tested strains, with the diameters of inhibition zones varied between 20 and 27 mm. Moreover, minimal inhibition and bactericidal concentration (MIC and MBC) values demonstrated that *T. capitatus* EOs generate the most important inhibitory effect against *E. coli* strains, with MIC values ranging from 0.02 to 0.78%. Concerning the mode of action of *T. capitatus* EO, the obtained data showed that treatment with this EO at its MIC reduced the viability of *E. coli* strains, their tolerance to NaCl and promoted the loss of 260-nm-absorbing material. In addition, in the presence of *T. capitatus* EO, cells became disproportionately sensitive to subsequent autolysis. Moreover, the inhibitory effect of *T. capitatus* was evaluated against two *E. coli* strains, experimentally inoculated (10^5^ CFU/g) in minced turkey meat, in the presence of two different concentrations of EO (MIC and 2 × MIC), and stored for 15 days. In both samples, EO exerted a bacteriostatic effect in the presence of concentrations equal to MIC. Interestingly, at 2 × CMI concentration, the bactericidal activity was pronounced after 15 days of storage. Our results highlighted that the use of essential oils, specially of *T. capitatus*, to inhibit or prevent the growth of extended-spectrum β-lactamase (ESBL)-producing *E. coli* in food, may be a promising alternative to chemicals.

## 1. Introduction

Generally, antimicrobials, such as antibiotics, are essential for treating infections caused by bacteria. However, their excessive use in veterinary and human medicine must be correlated with the emergency and spread of resistant bacteria, which render the treatment of infectious diseases in animals and humans ineffective, caused by the extended-spectrum β-lactamases (ESBLs)-producing bacteria [1]. The ESBLs-producing bacteria confer resistance to β-lactams antibiotics and to other classes of antibiotics, resulting in the expression of the multidrug-resistance (MDR) phenotype [2]. MDR isolates have escalated and become a significant cause of morbidity and mortality worldwide [3,4,5]. Resistant bacteria enter the food chain through animals, such as *Salmonella* via chickens, *E. coli* through raw milk and raw or undercooked ground meat, which can cause serious foodborne illness [3,4].

This unwanted change in the effectiveness of antibiotics and the loss of their important role as the primary agent in the treatment of infectious diseases has prompted researchers to find another alternative that can play the same role, with more benefits and less disadvantages. Recently, several studies have highlighted the different biological activities of aromatic and medicinal plants, in particular, their antifungal, antibacterial and antioxidant properties [6,7,8]. Thus, essential oils have been used in the field of food preservation, the perfume industry, cosmetics, pharmaceuticals and the food industry [9,10]. According to our knowledge, there are no available studies focusing on the antibacterial activity and the characterization of action mode of essential oils in Tunisia against ESBLs-producing *E. coli*.

In a previous investigation realized in our laboratory, the characterization of essential oil constituents was carried out. The data demonstrated that endemic plants from Tunisia had specific composition. This might suggest specific activity of essential oils obtained here. In view of these findings, we have hypothesized that Tunisian oils contain specific molecules that may act as antimicrobial agents.

The objective of the current investigation was to study the antibacterial activity of eight essential oils (*Eucalyptus globulus*, *Eucalyptus camaldulensis*, *Artemisia absinthium*, *Myrtus communis*, *Mentha pulegium*, *Trachyspermum ammi*, *Cymbopogon citratus*, and *Thymus capitatus*) against ESBLs-producing *E. coli,* isolated from meat. In addition, the characterization of the action of mode of two EOs (*T. ammi* and *T. capitatus*) was determined using four different assays (time–kill, lysis assays, loss of salt tolerance and integration of the cell membrane). Finally, the effect of the incorporation of two EOs (*T. ammi* and *T. capitatus*) in a cutlet of turkey meat on their microbiological qualities was assessed.

## 2. Results and Discussion

### 2.1. Antibacterial Effect of Essential Oils

Because of the problems of microbial resistance to synthetic antibiotics, several studies have focused on the antimicrobial power of EOs. In the current study, the antibacterial activity of eight essential oils (*E. globulus*, *E. camaldulensis*, *A. absinthium*, *M. communis*, *M. pulegium*, *T. ammi*, *C. citratus*, and *T. capitatus*) against extended-spectrum β-lactamase (ESBL)-producing *E. coli,* isolated from foods, was investigated. This antimicrobial potential was evaluated by determination of diameters of inhibition zones (IZ) (Table 1) and MIC and MCB values (Table 2).

As illustrated in Table 1, the obtained results show that the diameters of IZ generated by EOs varied significantly, according to the tested EO and evaluated *E. coli* strains. Generally, EO is considered active when the diameters of IZ engendered in its presence were greater than or equal to 15 mm [11]. Based on this condition, we can conclude that EOs of *T. capitaus*, *E.globulus*, *E. camaldulensis*, *T. ammi*, and *M. pulegium* exert an important antimicrobial effect against tested bacterial strains. In contrast, with inhibition diameters less than 15 mm, EOs of *A. absinthium*, *C. citratus* and *M. communis* show an intermediate activity.

Indeed, the analysis of obtained data demonstrated that EO of *T. capitatus* had the most important inhibitory effect against *E. coli* strains, since it represented the highest inhibition zone (27 mm) and the lowest MIC value (0.0975%) (Table 2). This remark is consistent with previous work carried out by Aouadhi et al. [6], which showed that *T. capitatus* is very effective against different bacterial species (*E. coli*, *S. typhimurium*, *S. aureus*, *P. aeruginosa*, *A. hydrophila*, *L. monocytogenes*, and *B. cereus*), with a maximum inhibition zone and MIC value varied between 17–40 mm and 0.025–0.4% (*v*/*v*), respectively.

Major components of *T. capitatus,* such as thymol, carvacrol, ocimene, and terpiene may be responsible for important antimicrobial activities. In fact, all of these compounds are well known for their antimicrobial properties. Indeed, several authors showed that EOs rich in phenolic derivatives, such as carvacrol and thymol, have strong antimicrobial activities [12,13]. In this same context, Dorman et al. [14] demonstrated that thymol had a broader spectrum of antibacterial activity against 25 different bacteria species. In addition, studies carried out by the World Health Organization highlighted that this constituent had strong antifungal and antibacterial activities against many species, including *Aspergillus* spp., *S. aureus* and *E. coli*. In fact, Lambert et al. [15] and Juven et al. [16] explained this phenomenon by the fact that thymol binds to membrane proteins and increases the permeability of the bacterial cell membrane. Other work suggested that this volatile compound is responsible for the inactivation of enzymes, including those involved in energy production and the synthesis of structural components [17].

Furthermore, the results in Table 1 and Table 2 signaled that EO of *T. ammi* had the lowest antimicrobial power compared to *T. capitaus*, with the IZ and MIC values in the range of 20 to 22 mm and 0.39 to 0.78% (*v*/*v*), respectively.

Concerning EO of *E. camaldulensis*, the obtained data showed that it is endowed with remarkable antimicrobial power against tested strains, with the IZ and MIC values varied between 16–18 mm and 0.0975–0.78% (*v*/*v*) respectively. Several studies have already demonstrated the antimicrobial activity of *Eucalyptus* compounds in different fields of life sciences [18,19]. These results are in agreement with those found by Farah et al. [20], who demonstrated that at a concentration of 0.2% (*v*/*v*), *E. coli*, *S. aureus* and *Aspergillus niger* are sensitive to EO of *E. camaldulensis*. According to Bouanoun et al. [21], the antimicrobial activity of *Eucalyptus* EO is attributed to eucalyptol or 1.8-cineole, which is a monoterpene belonging to the class of ethers. It had antioxidant and antibacterial properties and, therefore, explains the origin of its antimicrobial activity [22].

By comparing IZ diameters generated by *E. globulus* EO to that of *E. camaldulensis*, variability in their antibacterial effects against *E. coli* strains was observed. Indeed, IZ and MIC values of *E. globulus* were in the range of 15–18 mm and 0.195–0.78% (*v*/*v*), respectively. A study carried out in Morocco reported that IZ and MIC of *E. globulus* in the presence of *E. coli* and *S. aureus* were 48.15 and 13.50 mm and MICs of 0.15 and 0.75 mg/mL, respectively [18]. These results do not corroborate those obtained during this study, and this can be attributed to the chemical composition of our oil and to its origin.

The aromatogram produced with the *M. pulegium* EO demonstrates that *E. coli* is noticeably sensitive. Indeed, the potential inhibitors of the oil were in the range of 0.195–0.78% (*v*/*v*) and the IZ were in the order of 20–21 mm. These results are in agreement with those of Ghazghazi et al. [7], who studied the antimicrobial activity of *M. pulegium* against 10 microorganisms (*E. coli*, *S. typhimurium*, *S. aureus*, *P. aeruginosa*, *A. hydrophila*, *L. monocytogenes*, *B. cereus*, *Aspergillus niger*, *Aspergillus flavus*, and *C. albicans*). This study showed that the IZ and MIC values for the tested microorganisms were in the range of 15–30 mm and 0.05–0.8% (*v*/*v*), respectively. In addition, previous studies with the same plant, collected in Tunisia, Iran, Portugal and Morocco, demonstrated that these EO possess very important antimicrobial power, but there is great variation in the intensity of these activities against Gram-negative and Gram-positive bacteria. Such differences may be due to the chemical composition of EO. For example, Mahboubi and Haghi [23] showed that the volatile oil of Iranian *M. pulegium* had potent antimicrobial activity against Gram-positive bacteria, particularly against *L. monocytogenes,* where the inhibition zones were in the range of 8–21 mm, while the least susceptible bacteria were *E. coli* and *S. typhimurium* [7]. Our results confirm this previous work, which demonstrated significant antibacterial activities in the essential oil of mint in Algeria and, in particular, against *E. coli* [24].

As shown in Table 1 and Table 2, the results of the antimicrobial activity of EO of *A. absinthium*, showed an intermediate sensitivity of *E. coli*. Indeed, IZ were in the range of 11.33–13.67 mm. The antimicrobial power of *A. absinthium* has been previously reported by Riahi et al. [25], who showed that the EO of *A. absinthium,* observed in the Gafsa region, had a maximum inhibition zone of 11 mm and MIC value of around 25%, but for the Kasserine region, El Kef and Ghar Dimaou, the maximum inhibition zones and MIC values were around 14 mm and 12.5–25% (*v*/*v*) respectively.

Concerning the *M. communis* EO, it can be signaled that this oil can inhibit the microbial growth of *E. coli,* with IZ and MIC values in the range of 13.33 mm and 0.39–1.56% (*v*/*v*), respectively. This result is in agreement with the previous work of Chebaibi et al. [26], who revealed that the EO of *M. communis* had an antimicrobial activity against diverse bacterial species (*E. coli*, *B. subtilis*, *M. luteus* and *S. aureus*) when their growth was totally inhibited in the presence of 0.5% *v*/*v* of the EO. This antimicrobial activity is mainly due to the richness of the evaluated EO in a mixture of terpenols (α-terpineol and myrtenol) and 1,8-cineole. This fraction, which constitutes about 20% of the total oil, can be used in the food industry, as a means of prevention against pathogens that contaminate foodstuffs or in other pharmacological and medical fields [27].

The results obtained during the study of the antimicrobial activity of *C. citratus* EO were more or less average compared to other tested EOs. The maximum inhibition zones and MIC values were in the ranges of 12.33 mm and 0.78% (*v*/*v*), respectively. These results are in agreement with those obtained by Zulfa et al. [28], who showed that *C. citratus* EO can be used as an antimicrobial agent against *E. coli,* with a diameter inhibition zone and MIC values of 7.5 mm and 0.63% (*v*/*v*), respectively. Previous studies reported that *C. citratus* oil was more effective against Gram-positive bacteria, such as *S. aureus* and *B. cereus*. Based on the study of Bin et al. [29], it can be concluded that all of the Gram-negative bacteria have lipopolysaccharides on the outer layer and they have a negative charge all over the cell, which results in less affinity with the negative phenolic compounds. On the contrary Gram-positive bacteria have different structures and, for this, are perhaps more sensitive. The resistance of Gram-negative bacteria is attributed to their hydrophilic outer membrane, which can block the penetration of hydrophobic compounds into the target cell membrane [30,31].

Based on the obtained data, it can be signaled that the effect of different EOs against strains of *E. coli* used is low compared to that found in other studies in the presence of reference bacteria. These can be explained by two hypotheses. First, *E. coli* is a Gram-negative bacterium, so it is more resistant to the effects of antimicrobial agents than the Gram-positive ones. In this context, Dorman et al. [14] showed that Gram-positive bacteria exhibit IZ greater than those observed in Gram-negative bacteria. This can be explained by the fact that the cell wall structure of Gram-negative bacteria makes them less sensitive to the action of essential oils. The presence of an outer membrane, highly hydrophilic, constitutes a barrier to hydrophobic and amphiphilic macromolecules, which enter into the composition of these oils. Associated with the plasma membrane, which limits the passage of hydrophilic molecules, the intracellular penetration of all these compounds is considerably restricted in these types of bacteria [32]. This specific structure gives Gram-negative bacteria intrinsic resistance to most antibacterial molecules, including antibiotics, and these may explain the resistance of *E. coli,* which is a Gram-negative bacterium, observed during this study with certain EOs.

Second, the used isolates of *E. coli* are multi-antibiotic-resistant strains, capable of producing broad spectrum β-lactamase (ESBL)-type enzymes, which can destroy antibiotics prior to their penetration inside the cell or can modify the target of the antibiotic. The presence of this enzyme may explain the significant resistance of these strains to different Eos, in comparison to strains of the same species or different species.

### 2.2. Characterization of the Mode of Action in Essential Oils

The characterization of the action mode in two essential oils (*T. capitatus* and *T. ammi*) was carried out, during this study, for the first time. For this purpose, cell death and bacteriolysis experiments of seven strains of *E. coli,* multidrug resistant to antibiotics, were performed to measure the induced effects on cell viability.

#### 2.2.1. Time–Kill Studies

**Figure 1:**
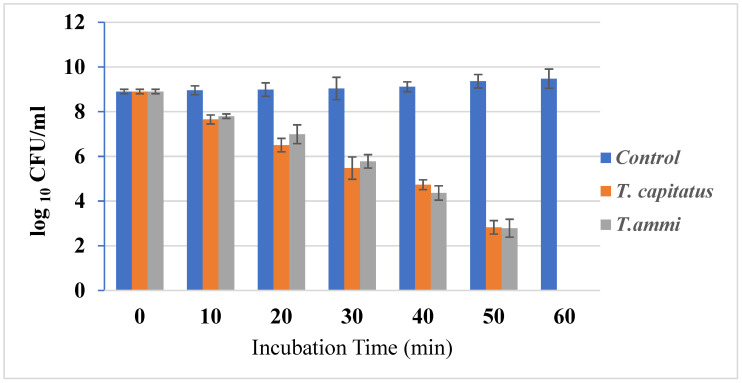
Time–kill curves *E. coli* cultures untreated and treated with the *T. capitatus* and *T. ammi* essential oils at concentrations corresponding to their MIC.

The growth of bacterial strains in the presence or absence of Eos, at concentrations equal to their MIC, was monitored (Figure 1). In fact, the growth of bacterial strains was inhibited after one hour of incubation in the presence of Eos, at concentrations equal to their MIC. This important effect could be due to the action of carvacrol in weakening and depolarizing the cytoplasmic membrane [33]. Due to their hydrophobic structure, this organ is an important cellular target for EO components [34]. Antimicrobial agents, such as thyme, cause whole cell lysis [35]. Further, in half an hour, carvacrol and thymol are structural isomers, having a phenolic hydroxyl placed in different locations on the phenolic ring. The hydroxyl group increases their hydrophilic capacity, which could help them dissolve in the microbial membrane and alter it [33].

#### 2.2.2. Cell Lysis Experiment

In order to study the lytic action of two EOs on the seven strains of *E. coli*, the absorbance of the bacterial strains, in the presence and absence of two studied Eos, at concentrations equal to their MIC, was evaluated. The results are expressed as the ratio of the absorbance measured at time T to the absorbance at 620 nm measured at time zero ((A620 (T)/A620 (T0)) × 100). From the obtained data, we can see that in the case of the control (absence of the two EOs), the absorbance is approximately 100% and, therefore, the absence of cell lysis is observed, while the presence of EOs led to a decrease in absorbance in all seven strains. In fact, the optical density decreased to 40% and 41%, respectively, in the presence of *T. capitatus* and *T. ammi* EOs.

Generally, some antimicrobial agents cause the irreversible destruction of the plasma membrane, resulting in cell death through a lytic process [36]. The present study showed that *T. capitatus* and *T. ammi* EOs exert a bacteriolytic effect against *E. coli* strains. In addition, the EO of *T. capitatus* exerts a greater lytic action. These results are consistent with those of Horne et al. [35], who showed that the EOs of oregano, rosewood and thyme generate lytic effects. However, other authors reported that plant extracts do not lyse bacterial cells but compromise the structural integrity of the plasma membrane and induce a loss of cytoplasmic material [37].

#### 2.2.3. Loss of Salt Tolerance

Regarding the tolerance of the strains to salts, the results are shown in Table 3. The addition of NaCl reduced the colony-forming ability. In fact, in the case of the control (untreated sample), we noticed that there was an increase in the percentage growth of the strains, which reached 100% for strain C930 at a concentration of 5%. Indeed, the sub-lethal lesion of the cell membrane of bacteria under the effect of essential oils induces the modification of its permeability and modifies its osmoregulation [38]. Therefore, loss of tolerance to salt or other toxic compounds can be exploited to reveal membrane damage in sub-lethally injured bacteria [39]. In addition, the treatment of our strains with both EOs significantly reduced the ability of bacterial growth on media containing NaCl.

#### 2.2.4. Integration of the Cell Membrane

The absorbance measurement is a new method that has allowed us to understand the mechanism of action in essential oils. Marked leakage of cytoplasmic material is considered indicative of gross and irreversible damage to the cytoplasmic membrane. Several antimicrobial agents, such as alpha-pinene and chlorhexidine, target the cell envelope, as well as compromising its integrity [36]. In fact, the effect of evaluated EOs on the absorbing material of *E. coli* strains at OD_260_ demonstrated that the OD_260_ of control suspensions was not significantly different after 30 min and 60 min (Table 4) but significant increases in the OD_260_ were observed after 60 min of treatment with both tested EOs. The EO of *T. capitatus* and *T. ammi* showed a release of absorbent constituents at 260 nm, suggesting that nucleic acids were lost through a damaged cytoplasmic membrane and, consequently, loss of permeability. 

### 2.3. Effect of Addition of Essential Oils against Strains of E. coli Inoculated into Turkey Meat

For food applications, concentrations of EO must be higher than those used *in vitro* antibacterial tests to achieve the same effect in food [34]. Thus, we chose two different concentrations of EOs of *T. capitatus* and *T. ammi* (MIC and 2 × MIC) to incorporate them into the minced turkey meat inoculated with *E. coli* strain C926, then studied their effects on the microbiological qualities of the meat during its conservation for 15 days.

#### 2.3.1. Effect of EOs on Total Germs

**Figure 2:**
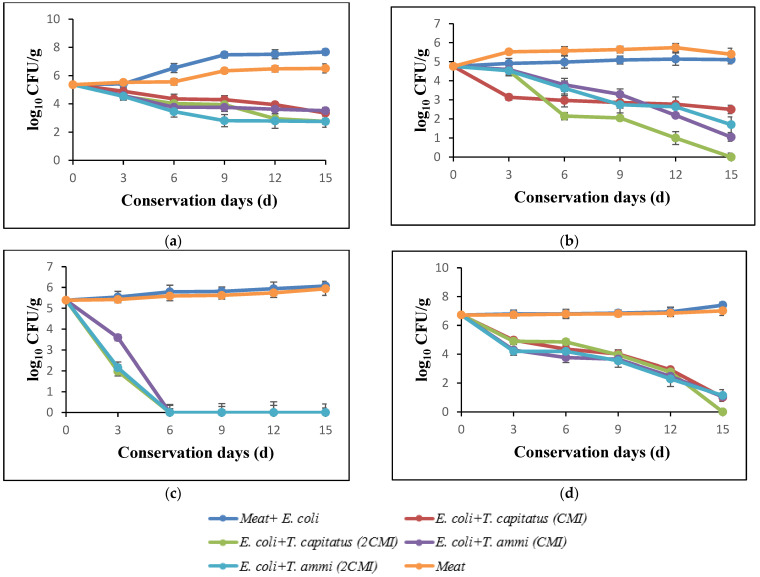
Effect of addition of essential oils against total germs (**a**), coliforms (**b**), E. *coli* (**c**), yeast and molds (**d**) into turkey meat.

The results of the evolution of total flora in turkey meat after storage at 4 °C for 15 days, in the presence and absence of Eos, are shown in Figure 2a. In the initial days, the loads of total flora corresponded to 5.47 log. In the absence of EOs, this count increased and reached 8.24 log after incubation for 15 days. In addition, following the meat treatment, a significant reduction according to the concentration of used EOs was observed. In fact, a bactericidal effect in the presence of *T. capitatus* EO at concentrations corresponding to 2 × MIC (0.04%) was noticed. Indeed, for the purpose of comparing bacterial growth in meat samples stored under aerobic conditions and bacterial growth in vacuum-packed samples, Tsigarida et al. [38,40] showed that the antibacterial activity of EOs, such as oregano oil, is more intense in the case of vacuum-packed samples.

#### 2.3.2. Effect of EOs on Yeasts and Molds

Figure 2d summarizes the effect of evaluated EOs on the yeasts and molds in turkey meat, after storage at 4 °C for 15 days. The obtained data shows that the bacterial growth decreases rapidly during the storage period, particularly in the presence of EO of *T. capitatus.* In fact, this intense antimicrobial activity is due to the high percentages of phenolic compounds, such as carvacrol and thymol [41].

#### 2.3.3. Effect of EOs on Total Coliforms

The antibacterial activity of *T. capitatus* and *T. ammi* EOs against total coliforms in turkey meat is shown in Figure 2b. After 12 days of storage, the load of total coliforms increased significantly, exceeding 6 log CFU/g in the case of the samples inoculated with *E. coli* (control) and that of meat only, whereas in the presence of EOs, the count decreased rapidly, reaching 3 log CFU/g in the presence of 2MIC of *T. capitatus* EO. These results are confirmed by El-Desouky et al. [42], who showed that the EOs of thyme and rosemary lead to a slight reduction in the development of total coliforms in minced meat.

#### 2.3.4. Effect of EOs on *Escherichia coli*

Figure 2c shows the antimicrobial power of EOs of *T. capitatus* and *T. ammi* against *E. coli* inoculated in turkey meat, without or with the addition of EOs. In fact, after 12 days of storage at 4 °C, an important inhibitory effect of both EOs at concentrations corresponded to 2MIC against *E. coli* strains artificially inoculated (10^8^ CFU/g) in minced turkey meat, while in the case of samples not treated with EO (control), an increase in the bacterial load of *E. coli* to almost 6 log during the 15 days of storage was observed. However, at MIC concentrations, *T. ammi* EO exerted a bacteriostatic effect after 12 days of storage. This in accordance with the work of Jayari et al. [9]; at low concentrations (between 0.01 and 0.05% (v/p)), EOs exert a bacteriostatic effect against *E. coli*, while at higher concentrations, the bactericidal effect takes place, especially for *T. capitatus* EO [9]. Thus, *T. capitatus* EO had a bioprotective effect and can, therefore, be used as a natural preservative ingredient in the food industry [43].

However, the in vitro antimicrobial activity of EO cannot be the same as in a food product. For foods rich in protein and fat, the effectiveness of EOs is less important because EOs bind to these compounds [44] or dissolve in the fatty phase of these products [45].

In addition, several factors can influence the antibacterial activity of Eos, such as temperature, storage conditions, pH or the composition of the food. Indeed, EO becomes more effective with the decrease in the pH of the food, the decrease in storage temperature or even the reduction in the amount of oxygen in the packaging [34]. Thus, the behavior of bacteria in the food ecosystem can be affected by intrinsic factors, such as the composition and extrinsic factors (temperature and oxygen limitation) of food products [44].

## 3. Material and Methods

### 3.1. Essential Oil

In order to research alternative antimicrobial agents to antibiotics, essential oils (*Eucalyptus globulus*, *Eucalyptus Camaldulensis*, *Artemisia vulgaris*, *Myrtus communis*, *Mentha pulegium*, *Trachyspermum ammi*, *Cymbopogon citratus*, *and Thymus Capitatus*) were tested for their antibacterial effect against extended-spectrum β-lactamase (ESBL)-producing *Escherichia* *coli* strains. The EOs were purchased from the company “Carthago Essences Sousse, Tunisia”.

### 3.2. Bacterial Strains and Growth Conditions

The strains used during this study were isolated on medium containing cefatoxime, as in a previous study carried out by Jouini et al. [3], from meats (Table 5). *E. coli* strains which showed a profile of multi-resistance to antibiotics were selected to study the antimicrobial effect of different EOs. These bacteria were maintained by sub-culturing on nutrient agars which are favorable to their growth for 24 h at 37 °C.

### 3.3. Evaluation of Antimicrobial Activity 

The antibacterial activity of investigated EOs against *E. coli* strains was evaluated using two methods. Firstly, the disc diffusion method was used to determine the qualitative antibacterial activity [46]. In fact, 100 μL of each strain (10^8^ CFU/mL) was spread on Muller Hinton agar plates. Sterile filter paper discs (6 mm in diameter) were separately impregnated with 15 μL of tested oil and placed on the agar which had previously been inoculated with the selected bacteria. Gentamicin (10 µg/disc) was used as a positive reference. Negative control corresponds to disc without sample. The inoculated plates were incubated first for 1 h at 4 °C and then for 24 h at 37 °C. The diameter of the growth-inhibition zone (including disc diameter of 6 mm) was used to estimate the qualitative antimicrobial activity of tested EOs.

The quantitative antibacterial effect of used EOs was determined using the broth dilution method as described by Aouadhi et al. [47]. This method was utilized to estimate the minimum inhibitory concentrations (MIC) and minimum bactericidal concentrations (MBC). Microbial growth was indicated by the presence of turbidity and a ‘pellet’ on the tube bottom. MIC was recorded visually as the lowest concentration in each row that completely inhibited bacterial growth. MBC is usually an extension from the MIC, where the microorganisms quantitatively indicate the minimum concentration as no viable organism appears in the culture [47].

### 3.4. Primary Mode of Action of Two EOs 

The mode of action of two EOs (*T. capitaus* and *T. ammi*) against *E. coli* was assessed using four tests.

#### 3.4.1. Time–Kill Studies

Time–kill studies allow one to characterize the antibacterial activity of two EOs by evaluating the reduction in bacteria count in the presence of extract at their MIC over several hours. In fact, the method described by Klepser et al. [48] and modified by Viljoen et al. [49] was used to evaluate the effect of *T. capitaus* and *T. ammi* EOs against *E. coli* strains by measuring the reduction in the number of CFU per milliliter over 24 h. The limit of quantification using this method is 10^2^ CFU [50]. 

#### 3.4.2. Bacteriolysis

The bacteriolysis assays of tested EOs against *E. coli* were assessed according to the standard method described by Carson et al. [37] and Guinoiseau et al. [50]. The results were expressed as a ratio (in percent) of the OD_620_ at each time point vs. the OD_620_ at 0 min.

#### 3.4.3. Loss of Cytoplasmic Material

The loss of cytoplasmic material was determined by measuring the release of 260-nm and 280-nm absorbing materials from *E. coli* strains according to method previously described by Carson et al. [37].

#### 3.4.4. Loss of Salt Tolerance 

The ability of *E. coli* cells treated with the tested EOs to grow on nutrient agar (NA) supplemented with NaCl was studied according to the method previously described by Carson et al. [37]. In fact, untreated and treated suspensions of *E. coli* for 30 min with *T. capitatus* and *T. ammi* at their MICs were plated into NA and NA containing NaCl at 5 to 100 g/L (NA-NaCl). After incubation at 37 °C for 24 h, the colonies were counted. The numbers of CFU per milliliter on each NA-NaCl plate were compared to those on the NA plate, and the result was expressed as a percentage.

### 3.5. Effect of Essential Oils against E. coli Inoculated in Meat

In the current study, the inhibitory effect of two EOs (*T. capitatus* and *T. ammi)* against 1 of among the 7 previously studied *E. coli* strains (C926) inoculated in minced turkey meat was investigated.

Fresh turkey meat obtained from a supermarket delivering meat products in Tunis (Tunisia) was transported to the laboratory of Pasteur Institute of Tunisia in an insulated cooler. Then, the samples were cut into small pieces using a sterile stainless steel knife and then chopped in a grinder. The minced meat was divided into portions of 100 g, packed in sterile polyethylene bags. The meat samples were inoculated with 10^5^ CFU *E. coli*/g of turkey meat. Then, the samples were treated with two different concentrations of *T. capitatus* or *T. ammi* EOs as summarized in Table 6 and homogenized in a stomacher for 5 min. Following this work, these samples were vacuum packed at the Higher School of Food Industries of Tunis, and stored at +4 °C for 12 days. Microbial analysis was performed according to standard methods [51] each 3 days. In fact, the plate count agar media were used to estimate the total viable count in meat samples. Plates were incubated for 72 h at 37 °C. Coliform counts were determined by plate method on violet red bile agar, prepared according to the manufacturer instructions. All plates were incubated at 37 °C for 24 h. Sabouraud agar was used for enumeration of yeasts and molds after incubation at 25 °C for 5 days. *E. coli* count was determined using Macconkey media after incubation at 37 °C for 24 h.

### 3.6. Statistical Analysis

All the results are expressed as mean ± standard deviation of three replications. Data were analyzed using statistical software. Data were subjected to one-way analysis of variance to evaluate the significance of each independent factor. A Fisher LSD test was used to discriminate the means with a significance level fixed at 5%. Calculations were performed using the SPSS20 program.

## Figures and Tables

**Table 1:** Zone of inhibition expressed in diameter (mm) of eight essentials oils against extended-spectrum β-lactamase (ESBL)-producing *E. coli*.

Strains	*Control*	*E. globulus*	*E. camaldulensis*	*A. absinthium*	*M. communis*	*M. pulegium*	*T. ammi*	*C. citratus*	*T. capitatus*
C930	20 ± 2.4	15 ± 1.73	21.33 ± 2.08	11.67 ± 0.58	12.67 ± 1.15	13 ± 2.65	21.33 ± 0.84	11 ± 0.58	25 ± 5
C923	21 ± 1.84	17.33 ± 1.03	20.33 ± 3.21	11.67 ± 1.15	12 ± 2	13.33 ± 1.15	20 ± 4.36	9.67 ± 1	25 ± 5
C920	21 ± 3.3	18 ± 2	20.67 ± 2.08	12.00 ± 1	13 ± 1	17 ± 5.29	21 ± 7.81	11 ± 1	27 ± 3.61
C924	23 ± 2.23	17.67 ± 1.53	20.33 ± 4.51	11.67 ± 1.15	11.67 ± 0.58	15 ± 1	21 ± 0.58	11.67 ± 1.53	24 ± 6.56
C926	22 ± 1.64	17 ± 1.73	20.67 ± 2.31	12 ± 2	13.33 ± 0.58	14 ± 1	21.66 ± 2.56	11.67 ± 0.58	22.33 ± 1.53
C921	23 ± 2.45	17 ± 1.73	20.67 ± 2.08	11.67 ± 1.15	11.67 ± 0.58	13 ± 1.73	20.67 ± 8.50	10.33 ± 0.38	23.67 ± 1.53
C928	22 ± 2.71	18.33 ± 0.58	21.33 ± 0.58	12.33 ± 0.58	12 ± 1	14.3 ± 1.15	22.67 ± 0.58	12.67 ± 1.53	25 ± 7

Values are means of three measurements. IZ diameters (mm) produced around the wells by adding 15 mL of EO.

**Table 2:** Minimum Inhibitory Concentrations (% *v*/*v*) (a) and Minimum Bactericidal Concentrations (% *v*/*v*) (b) of the tested essential oils.

Strains	Minimum Inhibitory Concentrations							
	*E. globulus*	*E. camaldulensis*	*A. absinthium*	*M. communis*	*M. pulegium*	*T. capitatus*	*T. ammi*	*C. citratus*
**C930**	0.78	0.39	0.78	1.56	0.39	0.0975	0.39	0.78
**C923**	0.78	0.39	0.78	0.78	0.39	0.0975	0.39	0.78
**C920**	0.39	0.78	0.78	0.78	0.39	0.0975	0.39	0.78
**C924**	0.39	0.78	0.78	0.195	0.195	0.0975	0.39	0.78
**C926**	0.195	0.39	0.78	0.39	0.78	0.0975	0.39	0.78
**C921**	0.78	0.78	0.78	1.56	0.195	0.0975	0.39	0.78
**C928**	0.78	0.78	0.78	0.39	0.39	0.0975	0.39	0.78
**Strains**	**Minimum Bactericidal Concentrations**							
	***E. globulus***	***E. camaldulensis***	***A. absinthium***	***M. communis***	***M. pulegium***	***T. capitatus***	***T. ammi***	***C. citratus***
**C930**	1.56	0.78	1.56	3.12	1.56	0.39	0.78	1.56
**C923**	1.56	0.78	1.56	0.78	1.56	0.39	0.78	1.56
**C920**	0.78	1.56	1.56	0.39	1.56	0.39	0.78	1.56
**C924**	0.39	0.78	1.56	0.78	0.39	0.39	0.78	1.56
**C926**	1.56	1.56	1.56	3.12	0.78	0.39	0.78	1.56
**C921**	1.56	1.56	1.56	0.78	1.56	0.39	0.78	1.56
**C928**	0.78	0.78	25	0.39	1.56	0.39	0.78	25

**Table 3:** Effect of various concentrations of NaCl on the growth of different strains of *E. coli* in the presence of two tested EOs at a concentration equal to their MIC.

Strains	Percentage of Strain Growth (%)								
	Control			*T. capitatus*			*T. ammi*		
	2.5%	5%	10%	2.5%	5%	10%	2.5%	5%	10%
**C920**	71.37	30.87	0	0	0	0	42.01	0	0
**C921**	100	82.23	60	68.62	0	0	0	0	0
**C923**	82.23	77.52	0	0	0	0	0	0	0
**C924**	82.3	81.19	0	50.3	0	0	70.12	0	0
**C926**	81.74	100	0	72.6	0	0	0	0	0
**C928**	81.74	98.21	0	0	0	0	0	0	0
**C930**	81.74	100	0	0	0	0	0	0	0

**Table 4:** Effect of EOs on the absorbing material of *E. coli* strains at OD_260_.

	Percentage of Initial OD (260)								
	Control			*T. ammi*			*T.capitatus*		
	0	30 min	60 min	0	30 min	60 min	0	30 min	60 min
C920	1	1.05 ± 0.011 ^a^	1.25 ± 0.01 ^a^	1	2.67 ± 0.01 ^c^	2.89 ± 0.02 ^d^	1	1.25 ± 0.016 ^a^	2.65 ± 0.02 ^c^
C921	1	1.03 ± 0.02 ^a^	1.2 ± 0.02 ^a^	1	2.4 ± 0.02 ^c^	2.9 ± 0.018 ^d^	1	1.5 ± 0.015 ^d^	2.8 ± 0.013 ^c^
C923	1	1.04 ± 0.03 ^a^	1.12 ± 0.01 ^a^	1	2.5 ± 0.01 ^c^	3.1 ± 0.017 ^d^	1	1.8 ± 0.021 ^d^	2.78 ± 0.013 ^c^
C924	1	1.04 ± 0.02 ^a^	1.21 ± 0.03 ^a^	1	2.58 ± 0.03 ^c^	2.9 ± 0.015 ^d^	1	1.87 ± 0.011 ^d^	2.7 ± 0.012 ^c^
C926	1	1.02 ± 0.01 ^a^	1.11 ± 0.02 ^a^	1	1.99 ± 0.02 ^b^	2.78 ± 0.022 ^c^	1	1.56 ± 0.015 ^d^	2.56 ± 0.021 ^c^
C928	1	1.01 ± 0.03 ^a^	1.17 ± 0.015 ^a^	1	1.98 ± 0.02 ^b^	2.54 ± 0.031 ^c^	1	1.98 ± 0.021 ^d^	2.67 ± 0.03 ^c^
C930	1	1.02 ± 0.02 ^a^	1.18 ± 0.005 ^a^	1	2.1 ± 0.015 ^c^	2.89 ± 0.016 ^d^	1	1.78 ± 0.018 ^d^	2.76 ± 0.017 ^c^
C920	1	1.01 ± 0.01 ^a^	1.2 ± 0.021 ^a^	1	2.3 ± 0.025 ^c^	3.2 ± 0.015 ^d^	1	1.67 ± 0.017 ^d^	2.79 ± 0.018 ^c^

Means with different letters are significantly different (*p* < 0.05).

**Table 5:** Origins of used bacterial strains [3].

Strains	Origin	b-Lactamase(s)
C923	Beef	CTX-M-1
C928	Chicken	SHV-5
C921	Chicken	CTX-M-8
C920	Beef	CTX-M-1. TEM-1b
C926	Turkey meat	CTX-M-1
930	Chicken	CTX-M-14. TEM-1b
C924	Beef	CTX-M-1

**Table 6:** Different experiment conditions.

Experiments	Conditions
1	Three 100 g portions of minced meat were inoculated with *E. coli*
2	Three 100 g portions of minced meat were inoculated with *E. coli* in the presence of MIC of *T. capitatus*
3	Three 100 g portions of minced meat were inoculated with *E. coli* in the presence of 2 × MIC of *T. capitatus* EO
4	Three 100 g portions of minced meat were inoculated with *E. coli* in the presence of 2 × MIC of *T. ammi* EO
5	Three 100 g portions of minced meat were inoculated with *E. coli* in the presence of 2 × MIC of *T. ammi* EO
6	portion of minced meat of 100 g

## Data Availability

Not applicable.

## Sample Availability

Samples of the compounds are available from the authors.
